# Case Report: Self-limiting penetrating cardiothoracic injury from a barbed shark dart involving the right atrium

**DOI:** 10.3389/fcvm.2026.1859460

**Published:** 2026-06-03

**Authors:** Xiong Tan, Weikai Dong, Yong Liu, Yinglong Lai, Jinjie Li

**Affiliations:** 1 Department of Cardiovascular Surgery, Affiliated Hospital of North Sichuan Medical College, Nanchong, Sichuan Province, China; 2 Cardiovascular Medical Center, Nanjing Drum Tower Hospital, The Affiliated Hospital of Nanjing University Medical School, Nanjing, Jiangsu Province, China; 3 Department of Surgery Center, Affiliated Hospital of North Sichuan Medical College, Nanchong, Sichuan Province, China

**Keywords:** barbed shark dart, cardiac tamponade, penetrating cardiac injury, right atrial injury, thoracic trauma

## Abstract

**Introduction:**

Penetrating thoracic trauma accounts for only 1%–13% of chest injury hospitalizations but is associated with high mortality due to involvement of the heart, great vessels, and lungs. Among these, foreign body injuries with retained objects are rare, and the role of specific structural features such as barbs in limiting injury severity has not been well described.

**Case presentation:**

A 54-year-old male sustained a penetrating injury to the right anterior chest from a barbed shark dart while fishing. On admission, he was relatively stable but with compensated hemodynamics. Echocardiography showed pericardial effusion with signs of early tamponade. Chest CT confirmed that the cylindrical foreign body had traversed the chest wall, the middle lobe of the right lung, and the pericardium, with the tip abutting the right atrial wall. Emergency median sternotomy revealed that the barbed shark dart had penetrated the right lung and pericardium and reached the right atrium. Notably, the backward-facing barbs at the posterior end of the dart prevented further forward migration, thereby averting perforation of the posterior right atrial wall and catastrophic bleeding. The foreign body was removed, the atrial puncture site was repaired, and a wedge resection of the injured lung was performed. The patient recovered uneventfully and remained asymptomatic at 1-year follow-up.

**Conclusion:**

This case highlights a unique “self-limiting” mechanism of penetrating cardiac injury, in which the barbed structure of the foreign body paradoxically reduced the extent of cardiac damage. Injury patterns in penetrating thoracic trauma depend heavily on the kinetic energy and structural characteristics of the object. For stable patients, a multidisciplinary approach involving imaging-guided surgical planning is critical.

## Introduction

Penetrating chest trauma, though accounting for only 1%–13% of all thoracic injury admissions (1), carries a high risk of mortality due to the potential for damage to critical structures such as the heart, great vessels, and lungs. The incidence of severe penetrating chest injuries is rising in some European metropolitan areas, from 1.4 to 3.5 per 100,000 inhabitants per year (2). Among these, penetrating cardiac injuries (PCI) are particularly devastating. While the overall survival rate for PCI has improved, it remains highly variable, with predictors including the mechanism of injury (stab vs. gunshot), hemodynamic status on arrival, and the specific cardiac structures involved (3, 4). Right ventricular involvement is relatively common (5), but injuries to any chamber can occur.

The diagnostic workup for hemodynamically stable patients has been revolutionized by multi-detector row computed tomography (MDCT), which offers high sensitivity and specificity for identifying pericardial effusion, active contrast extravasation, and the trajectory of a foreign body (6–8). For unstable patients or those with positive findings, prompt surgical exploration is the standard of care (9, 10). Multidisciplinary team (MDT) management, involving emergency, cardiothoracic, and trauma surgeons, is crucial for optimizing outcomes in such complex cases (9, 11).

The nature of the penetrating object is a key determinant of the injury pattern. Low-energy objects such as knives typically produce localized tract injuries, whereas high-energy projectiles such as bullets are associated with extensive cavitation and tissue destruction, as widely described in trauma literature (10, 12). However, the role of a projectile's unique physical features, such as barbs, is rarely discussed. We present a case of a penetrating right atrial injury from a barbed shark dart, where the barbs on the object played a paradoxical role in limiting the cardiac damage, ultimately contributing to the patient's survival.

## Case report

A 54-year-old male was accidentally stabbed in the right anterior chest by a barbed shark dart while fishing. He immediately presented to the emergency department of a local hospital. At that facility, only bedside echocardiography and chest X-ray were performed due to limited diagnostic capabilities. The local hospital lacked cardiac surgical capacity, and the patient was therefore transferred promptly to our hospital. On arrival, he reported right-sided chest pain and dyspnea of five hours’ total duration (including the initial evaluation and transfer time). Initial treatment with fluids and oxygen at the referring hospital had been unsuccessful.

On admission, the patient was conscious but tachypneic. Vital signs were: blood pressure 90/50 mmHg, heart rate 118 beats per minute, respiratory rate 28 breaths per minute, and oxygen saturation 94% on room air, indicating a compensated state.

**Figure 1:**
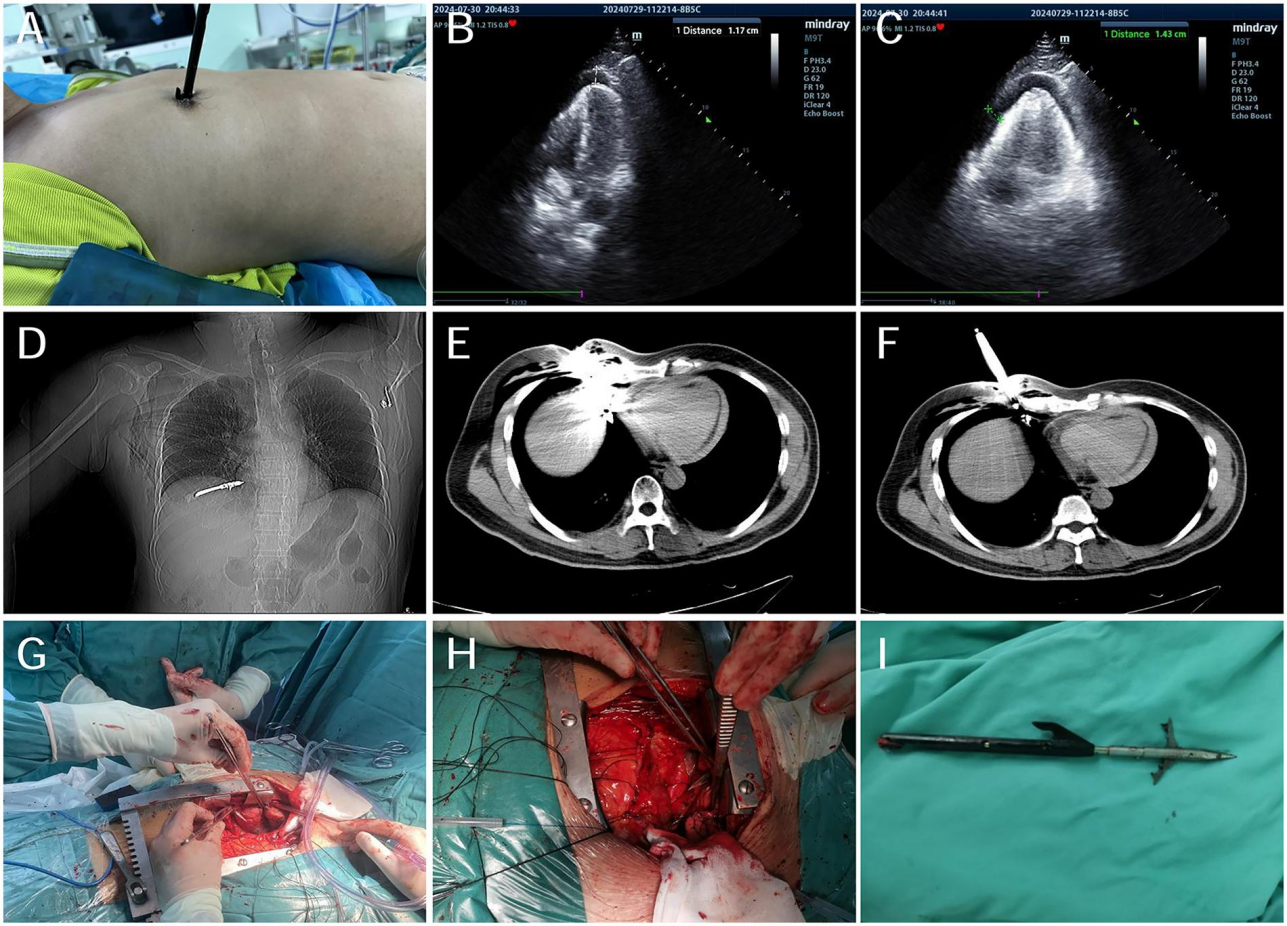
**(A)** physical examination revealed a cylindrical foreign body approximately 8 cm in length obliquely inserted into the right fourth intercostal space of the chest wall, close to the nipple area, that moved with respiration. **(B,C)** Echocardiography showed a fluid-filled dark area in the pericardial cavity, with a distance of 14.3 mm on the right ventricular side and 11.7 mm at the apex, suggesting cardiac tamponade. **(D–F)** Enhanced chest CT scan showed that the foreign body in the right thoracic cavity penetrated the chest wall, the middle lobe of the right lung tissue, and the pericardium, and the proximal end touched the right atrial wall. **(G–I)** During the procedure, the foreign body was identified as a cylindrical shark dart. The object had penetrated the chest wall, traversed the middle lobe of the right lung and the pericardium, and ultimately reached the right atrium. The shark dart was successfully removed by thoracotomy.

Physical examination revealed a cylindrical, barbed foreign body approximately 8 cm in length embedded obliquely in the right fourth intercostal space, close to the nipple area (Figure 1A). The object moved visibly with each breath. Breath sounds were diminished on the right side. Cardiac examination revealed distant heart sounds, but no murmurs or rubs. There were no signs of neck vein distension.

### Diagnostic assessment

Bedside echocardiography revealed a circumferential pericardial effusion, with a maximal separation of approximately 14 mm along the right ventricular free wall and 12 mm at the apex, accompanied by diastolic collapse of the right ventricle, consistent with cardiac tamponade (Figures 1B, C). A contrast-enhanced chest CT scan (Figures 1D–F) confirmed a large pericardial effusion and a right hemopneumothorax with approximately 10% lung collapse. The scan clearly delineated the trajectory of the dart: it had penetrated the chest wall, traversed the middle lobe of the right lung, and entered the pericardium. The proximal tip of the dart was seen abutting the lateral wall of the right atrium, though the exact plane of penetration was obscured by metallic artifact. No contrast extravasation was seen, suggesting a contained or non-active hemorrhage.

### Therapeutic intervention

Given the signs of tamponade and the CT findings, an emergency cardiac suture operation, combined with wedge resection of the middle lobe of the right lung, thoracotomy hemostasis, and closed thoracic and pericardial drainage were performed (Figures 1G–I). After successful anesthesia, the patient was placed in a supine position. The skin was routinely disinfected and draped. Through a pre-sternum incision, the sternum was longitudinally sawn open, and hemostasis was achieved by electrocoagulation before entering the thorax. A reverse T-shaped incision was used to open the pericardium, and the adhesions were loosened and the pericardium was suspended. During the operation, it was found that extensive subcutaneous emphysema was present on the right anterior chest wall. A sharp, inverted-spike-shaped foreign body, resembling a cone, could be seen penetrating the chest wall from the inner side of the areola and passing through the right lung lobe, then along the anterior side of the right diaphragmatic nerve and penetrating the pericardium into the right atrium. About 300 mL of dark black fluid could be seen in the pericardial cavity, and a small amount of dark red fluid, approximately 50 mL, was present in the right thoracic cavity. This fishhook-shaped foreign body penetrated the chest wall into the lung tissue and pericardial cavity, eventually reaching the right atrium. It moved with each breath, further causing damage to the lung tissue and the right atrium, and aggravating bleeding. However, the inverted spines at the tail prevented it from penetrating further, precisely avoiding a fatal cardiac perforation injury after penetrating the posterior wall of the heart.

Then, a 4-0 Prolene suture line was used around the puncture site of the right atrium, combined with a gauze pad for purse-string suturing. After removing the foreign body along the chest wall, the knot was tightened and tied, and protamine was used to neutralize heparin. Then, the right pleura was opened, and the damaged lung tissue was wedge-shaped resected using closed sutures. During the operation, the thoracic cavity was repeatedly rinsed with iodophor water and warm saline. The pneumoperitoneum test showed no obvious air leakage, and the rinsing fluid was drained. All the contamination and severely injured tissues on the chest wall wound were thoroughly removed, and about 2 mm of the skin margin was excised. A thorough debridement was performed, and the wound was rinsed with sterile normal saline and hydrogen peroxide alternately three times to achieve complete hemostasis. After checking that the gauze and instruments were correct, the chest was sutured layer by layer and closed. The majority of the pericardium was sutured with warm saline, and closed thoracic drainage tubes were placed in the pericardium and thoracic cavity. The sternum was closed with intermittent sutures using steel wire, and finally, the chest closure was completed with fishbone thread. After achieving thorough hemostasis and completing the wedge resection and atriorrhaphy, a closed thoracic drainage tube was placed in the right thoracic cavity and another in the pericardial cavity. The total blood loss after the operation was approximately 100 mL, and no blood transfusion was required during the operation.

Outcome and Follow-up: The patient was extubated on postoperative day 1 and transferred to the general ward on day 3. His postoperative course was unremarkable. He was discharged home on postoperative day 10. At his one-year follow-up, the patient was asymptomatic, had returned to his normal daily activities, and had no signs of late complications such as pericardial constriction or arrhythmia.

## Discussion

We report a case of a unique penetrating cardiac injury caused by a barbed shark dart. Our case is notable for two reasons: 1. The unusual mechanism of a self-limiting injury due to the foreign body's morphology; 2. the successful management using a multidisciplinary approach guided by modern imaging.

PCI are highly lethal. In an analysis of the National Trauma Data Bank by Asensio et al., the overall survival rate was approximately 33%, with gunshot wounds having a significantly worse prognosis than stab wounds (10% vs. 76% survival) (3). Our patient presented with a “stab-like” low-energy wound, which is associated with a better prognosis. However, the patient presented with cardiac tamponade. Some studies have identified tamponade as a key predictor of mortality (13); nevertheless, this view remains controversial. Other research has suggested that tamponade may paradoxically confer a protective effect by limiting further bleeding (14). The patient's survival aligns with data showing that timely surgical repair is the most critical factor for a positive outcome (15, 16).

Our case uniquely highlights the dual role of the penetrating object's physical characteristics. On one hand, the barbs of the shark dart caused significant tissue damage by lacerating the lung and anchoring into the chest wall, preventing easy removal and potentially causing more pain and bleeding (6). On the other hand, these same barbs acted as a natural “brake”, preventing the dart from completing its trajectory through the heart. A non-barbed projectile of the same length could have easily traversed the right atrium and exited the posterior wall, leading to a massive, likely fatal, through-and-through injury. This “self-limiting” mechanism, while rare, is a critical consideration for the operating surgeon. Understanding the foreign body's geometry before attempting removal can help anticipate complications and plan the appropriate surgical approach (e.g., having cardiopulmonary bypass ready, using a purse-string suture for control).

The use of MDCT was invaluable in this patient's management. As highlighted in recent articles, MDCT is the gold standard for assessing penetrating chest trauma in hemodynamically stable patients, providing crucial information about the trajectory, involved structures, and presence of active bleeding (7, 8). In our case, CT clearly demonstrated the dart's path through the lung and into the pericardium, abutting the right atrium. This allowed the surgical team to prepare for a complex cardiorrhaphy. Even with metallic artifact, the scan provided enough detail to rule out major vascular injury and plan the optimal incision (6). This is consistent with findings that CT has a negative predictive value of 99% in ruling out the need for surgical intervention in select patients (8). In resource-limited settings, even a non-contrast CT can be a powerful tool for surgical planning (17).

The surgical management of PCI has evolved. While the “cardiac box” concept is a useful clinical tool, its predictive value is limited, particularly for gunshot wounds (18). Therefore, a high index of suspicion and a low threshold for imaging or exploration are necessary. This case contributes to the growing evidence that management of retained intracardiac foreign bodies in hemodynamically stable patients should be individualized. In selected patients without tamponade or significant myocardial injury, conservative management with close echocardiographic follow-up may be a viable alternative (19). However, in the presence of cardiac tamponade or active bleeding, as in our patient, prompt surgical intervention remains necessary (20). Recent advances in minimally invasive surgery offer additional options for stable penetrating injuries (21), and the morphology of the FB—such as the barbed configuration in this case—can paradoxically limit injury severity and guide the decision between operative and non-operative strategies (22). Thus, clinical decisions must integrate hemodynamic status, imaging findings, and the unique physical characteristics of the penetrating object.

Our approach, combining a median sternotomy for maximal exposure with a simple purse-string technique for atriorrhaphy, is a standard and effective strategy (9, 10). The role of a multidisciplinary team cannot be overstated. The immediate collaboration between the emergency physicians, radiologists, anesthesiologists, and cardiac surgeons was essential for the seamless transition from diagnosis to definitive repair in this patient (11). This is particularly important in complex cases with multi-organ involvement (chest wall, lung, heart) (23).

The long-term prognosis for patients who survive a PCI is generally good if no severe neurological injury occurred. Our patient's excellent functional recovery at one year is consistent with other reports of isolated, repaired cardiac injuries (24, 25). Studies have shown that while the initial trauma can be devastating, survivors often achieve a good quality of life (26), especially when the injury is a low-energy, isolated wound without significant comorbidities.

This case has several limitations. It is a single case report, and the findings cannot be generalized. The unique morphology of the shark dart is not a common injury mechanism. The absence of intraoperative transesophageal echocardiography (TEE) might have missed subtle valvular or other intracardiac injuries, though the patient's excellent long-term outcome makes this unlikely. Finally, long-term psychological effects of such a traumatic event were not formally assessed.

In conclusion, we present a rare case of a surviving penetrating right atrial injury from a barbed shark dart. The barbs of the object, while contributing to tissue trauma, paradoxically prevented a more catastrophic cardiac injury. This case underscores the importance of rapid, MDT-based management, the critical role of MDCT in preoperative planning, and the need for the surgeon to consider the unique physical properties of a foreign body when planning its removal. Future research should continue to refine imaging protocols and develop predictive models, potentially leveraging artificial intelligence, to improve the risk stratification and management of patients with penetrating chest trauma (27).

## Data Availability

The original contributions presented in the study are included in the article/Supplementary Material, further inquiries can be directed to the corresponding author.
